# Comparative tissue response to a bovine peritoneum matrix and UltraPro mesh under experimental *Acinetobacter baumannii* contamination

**DOI:** 10.3389/fsurg.2026.1891844

**Published:** 2026-08-26

**Authors:** Ruslan Badyrov, Nurkassi Abatov, Alyona Lavrinenko, Nurlan Urazbayev, Elena Badyrova, Ilyas Bayakhmetov, Magauiya Abatov, Marina Izmailovich, Yevgeniy Kamyshanskiy

**Affiliations:** 1 Department of Surgery, Karaganda Medical University, Karaganda, Kazakhstan; 2 Head of the Scientific Research Laboratory of the Institute of Life Sciences, Karaganda Medical University, Karaganda, Kazakhstan; 3 Clinic of the Medical University, Karaganda Medical University, Karaganda, Kazakhstan; 4 Department of Internal Medicine, Karaganda Medical University, Karaganda, Kazakhstan

**Keywords:** abdominal wall reconstruction, acinetobacter baumannii, biologic extracellular matrix, hernia repair, histomorphometry, postoperative contamination, prosthetic mesh

## Abstract

**Background/objectives:**

Biologic extracellular matrix (ECM) implants are investigated for abdominal wall reconstruction because they may support tissue remodeling while limiting persistent foreign-body reactions. However, their response to contamination with multidrug-resistant pathogens remains insufficiently characterized. This exploratory study compared a bovine-derived peritoneum ECM scaffold with UltraPro mesh in a rat model of *Acinetobacter baumannii* contamination.

**Methods:**

Thirty outbred rats were included. Twenty-four animals underwent ECM or UltraPro implantation and were evaluated on postoperative days 10 and 20 (*n* = 6 per subgroup); six non-implanted animals receiving sterile saline served as a non-implanted reference group. Abdominal wall defects were reconstructed and inoculated with 200 µL of an *A. baumannii* suspension at 10^9^ CFU/mL. Tissue responses were assessed using semi-quantitative histological scoring based on ISO 10993-6 principles. Conventional microbiological culture was also performed.

**Results:**

All animals survived, and no clinically evident wound infection, dehiscence, or abscess formation was observed. On day 10, ECM demonstrated greater necrosis and polymorphonuclear-cell infiltration than UltraPro. By day 20, ECM showed reduced necrosis, increased cellular colonization, and greater reticulin fiber deposition, consistent with early tissue organization. UltraPro demonstrated increasing multinucleated giant-cell activity, fibrosis, granulomatous inflammation, and capsule formation. Conventional culture did not recover viable *A. baumannii* from ECM specimens, whereas the organism was recovered from 2 of 12 UltraPro specimens.

**Conclusions:**

Under experimental *A. baumannii* contamination, ECM and UltraPro demonstrated distinct early patterns of tissue response. ECM showed a more pronounced acute inflammatory response followed by histological changes consistent with early tissue organization, whereas UltraPro demonstrated progressive foreign-body and fibrotic changes during the observation period. Because of the small subgroup size, short follow-up, absence of concurrent sterile implant controls, semi-quantitative histological assessment, and lack of quantitative microbiological and biomechanical evaluation, these findings should be considered exploratory and do not establish superior functional or clinical performance of either material. Longer-term microbiological, mechanical, and functional studies are required.

## 1 Introduction

Abdominal wall reconstruction remains a clinically important surgical challenge, particularly when repair is performed in contaminated or potentially contaminated surgical fields. Reconstruction of abdominal wall defects relies on a wide spectrum of prosthetic materials, including permanent synthetic meshes, partially absorbable composite devices, and biologic scaffolds derived from decellularized extracellular matrix (ECM). Biologic ECM implants preserve collagen-rich three-dimensional architectures that may support cell infiltration, neovascularization, and gradual remodeling while reducing the persistence of chronic foreign-body reactions commonly associated with nondegradable synthetic materials (1–3). Their biological behavior is determined by their biochemical composition, microstructural integrity, degree of decellularization, and preservation of native ECM components.

Implant-associated infection remains one of the most significant challenges in abdominal wall reconstruction, particularly in contaminated surgical fields or in the presence of multidrug-resistant organisms. Mesh infection contributes to prolonged hospitalization and impaired wound healing and may require implant removal or revision surgery (4, 5). Similar difficulties related to biomaterial-associated infection have been reported in other surgical disciplines, including orthopedic implant surgery, where persistent infection substantially complicates postoperative management (6). Although biologic scaffolds have frequently been proposed for use in contaminated conditions, the available clinical and experimental evidence remains heterogeneous. Recent randomized clinical trials have challenged the assumption that biologic mesh is clinically superior in contaminated ventral hernia repair, with synthetic mesh demonstrating lower recurrence rates and comparable safety outcomes in some studies (7, 8). These findings underscore the need for mechanistic studies evaluating tissue–material interactions under a standardized microbial challenge.

Synthetic meshes, including polypropylene-containing materials such as UltraPro, provide durable reinforcement and standardized handling characteristics. However, under contaminated conditions, synthetic implants may be susceptible to bacterial adhesion and biofilm formation and may be associated with persistent inflammation, fibrosis, and chronic foreign-body reactions, potentially impairing tissue integration and contributing to postoperative complications (2, 5).

*Acinetobacter baumannii (A. baumannii)* is a resilient nosocomial pathogen capable of prolonged survival on abiotic surfaces, biofilm formation, and acquisition of multidrug resistance. It is an important cause of postoperative wound infections and device-associated complications (9, 10). Its ability to persist in the hospital environment, adhere to implanted biomaterials, evade host defense mechanisms, and develop multidrug resistance makes it particularly relevant to implant-associated infection. Regional data from Kazakhstan have documented carbapenem-resistant A. baumannii strains and the circulation of internationally distributed epidemic clones (11, 12). Clinically, A. baumannii infections are associated with prolonged hospitalization, increased treatment complexity, and unfavorable outcomes, particularly among surgical patients and those with device-associated infections (13–15). Experimental studies have also demonstrated the ability of A. baumannii to induce inflammatory responses and persist on biomaterial surfaces, potentially complicating implant integration (16, 17).

The interaction between bacterial contamination, implant characteristics, and the host tissue response may substantially influence inflammation, fibrosis, foreign-body reactions, and biomaterial integration. Experimental models provide a controlled framework for investigating these early biological events at the implant–tissue interface. However, findings from short-term small-animal studies should be interpreted cautiously because they cannot establish long-term remodeling, mature capsule formation, biomechanical durability, recurrence risk, or clinical superiority of one implant material over another.

The aim of this exploratory study was to compare the early histological and histomorphometric tissue responses to a bovine-derived peritoneum ECM scaffold and synthetic UltraPro mesh in a standardized rat model of *A. baumannii* contamination. The study focused on necrosis, inflammatory-cell infiltration, multinucleated giant-cell activity, fibrosis, neovascularization, matrix colonization, and early tissue organization. Biomechanical and functional outcomes were outside the predefined scope of the study.

## 2 Materials and methods

### 2.1 Study design and ethical approval

A controlled exploratory *in vivo* study was conducted to evaluate the histological, histomorphometric, and microbiological tissue responses to a bovine-derived peritoneum extracellular matrix (ECM) implant and synthetic UltraPro mesh under standardized *A. baumannii* contamination. All experimental procedures were performed at the vivarium of Karaganda Medical University in accordance with the ARRIVE 2.0 guidelines, the U.K. Animals (Scientific Procedures) Act 1986, Directive 2010/63/EU, and institutional policies on laboratory animal welfare. The study protocol was approved by the Local Bioethics Committee of Karaganda Medical University (Protocol No. 3, 27 February 2024). Thirty sexually mature outbred albino rats of both sexes were included. Sex distribution was recorded for descriptive purposes; the study was not powered for sex-stratified analysis.

### 2.2 Animals and experimental groups

Thirty outbred albino rats of both sexes, weighing 180–220 g, were housed under standard vivarium conditions at a temperature of 20–24°C and a 12 h light/dark cycle, with free access to food and water. Animals were randomly allocated to two implantation groups, ECM and UltraPro, and each group was subdivided according to the postoperative assessment time point of 10 or 20 days. Each implanted subgroup included six animals (*n* = 6). An additional non-implanted saline control group (*n* = 6) was included to document baseline tissue morphology and identify nonspecific histological changes unrelated to implantation or bacterial contamination. These animals received sterile 0.9% saline and were euthanized on postoperative day 10 because the group was intended solely for baseline histological assessment. This group was not a sterile implant control and therefore could not be used to distinguish material-specific effects from the effects of bacterial contamination. No concurrent non-contaminated ECM or UltraPro implantation groups were included in the present experimental design. The experimental groups, implant materials, contamination conditions, and observation time points are summarized in Table 1.

**Table 1:** Experimental groups included in the primary comparative analysis.

Group	Inoculum/condition	Implant material	Observation period	*n*
1	*A. baumannii*, 10^9^ CFU/mL	ECM	10 days	6
2	*A. baumannii*, 10^9^ CFU/mL	ECM	20 days	6
3	*A. baumannii*, 10^9^ CFU/mL	UltraPro mesh	10 days	6
4	*A. baumannii*, 10^9^ CFU/mL	UltraPro mesh	20 days	6
5	Sterile 0.9% NaCl	No implant	10 days	6

Separate non-contaminated histomorphometric observations obtained after implantation of the same bovine-derived xenoperitoneal ECM were included as descriptive contextual data. These observations originated from an independent experimental series conducted under the same animal housing conditions and using the same histomorphometric methodology, with assessment on postoperative days 7, 14, and 21. Because they were obtained in a separate experiment, at different observation time points, and without a corresponding non-contaminated UltraPro group, these data were not considered concurrent controls and were not included in the primary comparative statistical analysis. They are presented solely to provide descriptive context regarding the temporal tissue response to xenoperitoneal ECM implantation in the absence of bacterial contamination.

Postoperative days 10 and 20 were selected to characterize early tissue responses to the implanted materials under standardized bacterial contamination. These time points permit assessment of acute and subacute inflammatory-cell infiltration, early tissue integration, and the transition toward connective tissue organization (18, 19).

### 2.3 Bacterial strain and inoculum preparation

A multidrug-resistant strain of *A. baumannii* was obtained from the microbiological collection of the Shared Resource Laboratory of Karaganda Medical University. Species identification was confirmed by matrix-assisted laser desorption/ionization time-of-flight mass spectrometry (MALDI-TOF MS), and antimicrobial susceptibility testing was performed in accordance with Clinical and Laboratory Standards Institute (CLSI) guidelines. For inoculum preparation, the strain was subcultured on nutrient agar and incubated for 18–24 h at 37 °C. Fresh colonies were suspended in sterile 0.9% saline, and the bacterial concentration was adjusted to 10^9^ CFU/mL using spectrophotometric calibration at an optical density of 600 nm (OD_600_). The final inoculum concentration was confirmed by serial dilution and colony counting. Immediately after implantation, 200 µL of the bacterial suspension was applied directly to the implant surface, corresponding to a total inoculum of 2 × 10^8^ CFU per animal.

### 2.4 Surgical procedure, anesthesia, and perioperative care

General anesthesia was induced with isoflurane in an inhalation chamber at a concentration of 4%–5% and maintained at 1.5%–2.5% in oxygen. Adequate anesthetic depth was confirmed by the absence of withdrawal reflexes. The abdominal wall was shaved and disinfected with 70% ethanol followed by povidone–iodine. All surgical procedures were performed under aseptic conditions. A standardized anterolateral abdominal wall defect was created without entering the peritoneal cavity. Each defect was reconstructed using an implant measuring 2.5 × 1.5 cm. The bovine-derived xenoperitoneal ECM used in this study had been developed and characterized in previous preclinical investigations (20). The ECM scaffold or UltraPro mesh was fixed to the muscle layer in an edge-to-edge fashion using 4-0 Vicryl sutures on an atraumatic needle. After implantation, 200 µL of the *A. baumannii* suspension at 10^9^ CFU/mL was applied directly to the implant surface. Postoperative analgesia consisted of meloxicam administered subcutaneously at 1–2 mg/kg once daily for 2 days. Animals were monitored continuously until recovery from anesthesia and were subsequently examined daily until the scheduled experimental endpoint. Clinical monitoring included general condition, activity, hydration status, food and water intake, body weight, and postoperative wound status. Wounds were assessed for hyperemia, edema, discharge, suture-line integrity, wound dehiscence, and macroscopically detectable abscess formation. Clinical observations were recorded descriptively. No formal wound-infection scoring system or validated pain/distress scoring scale was used.

### 2.5 Tissue collection and processing

On postoperative days 10 or 20, animals were euthanized by CO_2_ inhalation followed by cervical dislocation in accordance with institutional guidelines. The implant and surrounding abdominal wall tissues were excised together with standardized 5-mm margins from the implant–tissue interface. Samples intended for histological examination were fixed in 10% neutral buffered formalin (pH 7.0) at 4 °C for 24 h. Implant-associated tissue samples were additionally collected under aseptic conditions for conventional microbiological culture. The presence of viable *A. baumannii* and other cultivable microorganisms was assessed using standard culture methods. Recovered microorganisms were identified by standard microbiological procedures, and bacterial growth was recorded using semiquantitative culture categories. Quantitative bacterial burden expressed as CFU per gram of tissue, bacterial DNA quantification, and direct biofilm assessment by scanning electron microscopy, confocal microscopy, or other imaging methods were not performed. Accordingly, microbiological findings were interpreted only as culture-based evidence of viable bacterial recovery under the applied conditions and not as evidence of complete bacterial clearance, absence of biofilm, or antimicrobial activity of either implant material.

### 2.6 Histological preparation

After fixation, tissues were washed, dehydrated through graded ethanol (70%–100%), cleared in xylene, and embedded in paraffin. Sections (3–4 µm thick) were prepared using a Thermo Scientific HM 340E microtome. Histological staining was performed as follows: hematoxylin and eosin (H&E) for assessment of general morphology and inflammatory infiltrates; Masson's trichrome stain (Bio-Optica, Italy) for visualization of collagen fibers; Gomori silver stain (Bio-Optica, Italy) for reticulin (type III collagen) fibers; and Weigert's fuchsin–resorcin stain for evaluation of elastic fibers in UltraPro specimens.

Preparation and decellularization of the xenoperitoneal biomaterial were performed according to a previously developed and patented protocol (21).

Microscopy was performed using a Zeiss Axio Lab 4.0 microscope at magnifications of ×40, ×100, and ×200. Images were captured using AxioVision 7.2 software.

### 2.7 Histomorphometric evaluation

Tissue reactions were evaluated semi-quantitatively according to ISO 10993-6 criteria for local effects after implantation, supplemented by an adapted scoring system previously applied in experimental studies of xenoperitoneal biomaterials under bacterial contamination. All histological evaluations were performed by a histopathologist blinded to implant type and observation period. Each applicable parameter was evaluated using a semi-quantitative four-point scale adapted from ISO 10993-6 criteria and previous experimental studies of xenoperitoneal biomaterials. Scores were assigned as follows: 1 = minimal changes (isolated or weak focal findings without substantial tissue disruption); 2 = moderate changes (limited focal or multifocal findings involving part of the implantation area); 3 = marked changes (extensive findings involving a substantial portion of the implant–tissue interface); and 4 = severe changes (diffuse or extensive alterations associated with pronounced inflammation, necrosis, fibrosis, or tissue disorganization). Granuloma formation was additionally recorded as *n* (%) (22, 23). Although based on established ISO 10993-6 principles, the modified scoring approach has not undergone independent validation for contaminated extracellular matrix implantation models and should therefore be regarded as a semi-quantitative exploratory assessment tool.

Fibrosis, reticulin fiber deposition, and neovascularization were assessed using the predefined semi-quantitative scoring system. Quantitative digital measurements of collagen area fraction, collagen density, fiber orientation, and vascular density were not included in the original experimental protocol and were not performed. The available histological images were acquired primarily for semi-quantitative pathological evaluation and illustrative purposes. Standardized whole-slide acquisition, uniform field selection, image calibration, and a prospectively defined digital morphometry protocol were not used. Therefore, retrospective quantitative analysis of the available representative micrographs was not performed because it could introduce selection and measurement bias.

### 2.8 Statistical analysis

Statistical analysis was performed using IBM SPSS Statistics version 26.0 (IBM Corp., Armonk, NY, USA). Because the histomorphometric outcomes were measured using an ordinal semi-quantitative scoring system and each subgroup included six animals, nonparametric methods were applied. Comparisons between ECM and UltraPro at the same observation time point and comparisons between postoperative days 10 and 20 within the same implant group were performed using the Mann–Whitney *U*-test. Data are presented as median [interquartile range]. Exact two-sided *p*-values are reported whenever available, and *p* < 0.05 was considered statistically significant. Because multiple histomorphometric endpoints were evaluated, the Benjamini–Hochberg false discovery rate (FDR) procedure was applied to reduce the risk of type I error. For the principal comparisons, both unadjusted *p*-values and FDR-adjusted q-values are reported. An FDR-adjusted q-value < 0.05 was considered statistically significant after correction for multiple comparisons.

Sample size was determined during study planning using the resource equation approach proposed by Arifin and Zahiruddin (24), which is applicable to exploratory animal studies when reliable preliminary estimates of effect size and variance are unavailable (24). For the present design, comprising two implant groups and two observation time points, the recommended sample size was 4–6 animals per subgroup. Six animals were therefore included in each subgroup, corresponding to the upper limit of the recommended range. Because animals were euthanized at each predefined time point, the sample size was applied independently to each subgroup.

No formal *a priori* power calculation based on an expected effect size was performed. Accordingly, the study may have had insufficient statistical power to detect small or moderate differences. Non-significant findings were not interpreted as evidence of equivalence or absence of a biologically relevant effect. Effect sizes and confidence intervals were not calculated, and the statistical findings should therefore be regarded as exploratory and hypothesis-generating.

## 3 Results

Histomorphometric findings for the contaminated implantation groups are summarized in Table 2. A total of 24 contaminated implanted specimens, including 12 ECM and 12 UltraPro specimens, and six non-implanted saline control specimens were processed. The primary comparative analysis included the contaminated ECM and UltraPro groups assessed on postoperative days 10 and 20. Separate histomorphometric observations from an independent non-contaminated xenoperitoneal ECM experiment are presented in Table 3. These data were included solely as descriptive contextual information and were not incorporated into the primary statistical analysis. Because they originated from a separate experimental series, were obtained at different observation time points, and did not include a corresponding non-contaminated UltraPro group, they should not be regarded as concurrent control data. Therefore, these observations cannot be used to estimate the independent effect of bacterial contamination or to compare sterile tissue responses between ECM and UltraPro.

**Table 2:** Histomorphometric characteristics of tissue responses following ECM and UltraPro implantation under *A. baumannii* contamination.

Parameter	ECM, Day 10	ECM, Day 20	UltraPro, Day 10	UltraPro, Day 20
Necrosis	3.0 [0.75]	2.0 [0.0]	1.0 [0.0]^b^	1.0 [0.0]^b^
Implant degradation	2.0 [0.75]	1.0 [0.0]	1.0 [0.0]	1.5 [1.0]
Polymorphonuclear cells	2.5 [1.0]	2.0 [0.0]	1.0 [0.0]^b^	1.0 [0.0]^b^
Lymphocytes	1.0 [0.0]	2.0 [0.75]^a^	2.0 [0.0]	2.0 [0.0]
Multinucleated giant cells	1.0 [0.0]	1.0 [0.0]	1.0 [0.0]	2.5 [1.0]^a^^,^^b^
Neovascularization	1.0 [0.0]	1.0 [0.0]	2.0 [0.75]^b^	2.0 [0.0]^b^
Fibrosis	1.0 [0.0]	1.0 [0.0]	2.0 [1.0]^b^	3.0 [0.75]^b^
Granuloma formation, n/N (%)	0/6 (0%)	0/6 (0%)	2/6 (33.3%)	5/6 (83.3%)
Cellular colonization of the matrix	1.5 [1.0]	3.0 [1.0]^a^	–	–
Fat infiltration	–	–	2.0 [0.75]	3.0 [0.75]
Capsule formation	–	–	1.0 [0.0]	2.5 [1.0]
Phagocytosis	–	–	1.0 [0.0]	1.5 [1.0]
Reticulin fibers	1.0 [0.0]	2.0 [0.75]^a^	1.0 [0.0]	1.0 [0.0]^b^
Elastic fibers	–	–	1.0 [0.75]	1.5 [1.0]

Data are presented as median [interquartile range], unless otherwise indicated. Histomorphometric parameters were evaluated using a four-point semi-quantitative scale: 1 = minimal changes, 2 = moderate changes, 3 = marked changes, and 4 = severe changes. Granuloma formation is presented as n/N (%).

a FDR-adjusted *q* < 0.05 compared with postoperative day 10 within the same implant group.

b FDR-adjusted *q* < 0.05 compared with the ECM group at the corresponding postoperative time point.

Exact unadjusted *p*-values and Benjamini–Hochberg FDR-adjusted q-values for the principal comparisons are presented in Table 5. The symbol “–” indicates that the parameter was not applicable or was not assessed for the corresponding implant material.

ECM, extracellular matrix; FDR, false discovery rate; IQR, interquartile range.

**Table 3:** Reference histomorphometric characteristics of non-contaminated xenoperitoneal ECM implantation at representative time points.

Implant/time point	Granulocytes	Lymphocytes	Plasma cells	Macrophages	Fibroblasts/fibrocytes	Angiogenesis	Connective tissue maturity	Vessel characteristics
Xenoperitoneal ECM, 7 days	102.0 [87.8–108.5]	103.0 [91.5–106.3]	13.5 [11.5–15.3]	29.5 [25.8–30.3]	61.0 [55.3–64.5]	8.0 [7.0–9.3]	Focal mature connective tissue	Thin-walled congested vessels; isolated swollen endothelial cells
Xenoperitoneal ECM, 14 days	37.5 [30.0–58.3]	125.0 [111.3–127.5]	9.5 [6.0–11.3]	32.0 [28.8–35.3]	93.5 [89.5–102.3]	12.5 [8.8–14.0]	More mature connective tissue formation	Congested vessels with developing muscular wall
Xenoperitoneal ECM, 21 days	14.5 [9.8–16.0]	55.5 [51.3–64.3]	2.0 [1.0–4.5]	27.5 [21.8–35.0]	195.5 [179.8–207.8]	9.0 [8.0–11.3]	Marked connective tissue maturation	More organized vascular structures

Data are presented as median [IQR]. Cellular parameters were assessed per 300 cells at ×400 magnification.

Data are presented as median [IQR]. These observations were obtained from an independent experimental series on postoperative days 7, 14, and 21. They are provided solely as descriptive contextual information and were not included in the primary statistical analysis. They should not be regarded as concurrent control data and cannot be directly compared with the contaminated ECM or UltraPro groups. ECM, extracellular matrix; IQR, interquartile range.

### 3.1 Clinical observations

All animals survived until the scheduled experimental endpoints, and no unexpected mortality or severe postoperative complications occurred. The general condition, activity, hydration status, and food and water intake remained satisfactory throughout the observation period. Body weight showed an overall increasing trend, and no clinically relevant body-weight deficit was identified.

No clinically evident surgical-site infection, wound dehiscence, disruption of the suture line, or macroscopically detectable abscess formation was observed in any animal subjected to experimental *A. baumannii* contamination. Because clinical observations were recorded descriptively and no formal wound-infection or pain/distress scoring system was used, numerical comparisons of these outcomes between the experimental groups were not performed.

### 3.2 Microbiological findings

Conventional microbiological culture did not recover viable *A. baumannii* from any ECM-associated specimen (0/12). In contrast, viable *A. baumannii* was recovered from 2 of 12 UltraPro-associated specimens (16.7%). These findings were considered descriptive because the study was not designed or powered for a definitive comparison of microbiological outcomes. A summary of post-explant culture findings, including recovery of the inoculated *A. baumannii*, non-target microorganisms, semiquantitative bacterial growth, and culture-negative specimens, is presented in Table 4.

**Table 4:** Post-explant microbiological culture findings following ECM and UltraPro implantation under experimental *A. baumannii* contamination.

Implant	Observation time	*n*	*A. baumannii* recovered, *n* (%)	Non-target microorganisms recovered^a^	Semiquantitative bacterial growth (positive cultures)	No bacterial growth, *n* (%)
ECM	Day 10	6	0 (0%)	*Klebsiella pneumoniae, Proteus vulgaris, Proteus mirabilis*	10^5^–10^7^ CFU	0 (0%)
ECM	Day 20	6	0 (0%)	*Lactobacillus* spp., *Staphylococcus aureus, Klebsiella pneumoniae, Enterobacter cloacae, Klebsiella aerogenes*	10^4^–10^6^ CFU	1 (16.7%)
UltraPro	Day 10	6	1 (16.7%)	*Enterobacter cloacae, Escherichia coli*	10^5^–10^6^ CFU	2 (33.3%)
UltraPro	Day 20	6	1 (16.7%)	*Klebsiella oxytoca, Enterobacter cloacae, Staphylococcus aureus, Lactobacillus* spp.	10^5^–10^7^ CFU	1 (16.7%)

ECM, extracellular matrix; CFU, colony-forming units. Semiquantitative bacterial growth is presented as the observed range among culture-positive specimens within each subgroup. Microorganisms other than the inoculated *A. baumannii* strain were classified as non-target isolates and were considered incidental microbiological findings. Conventional culture was used to detect viable bacteria and does not provide information regarding total bacterial burden or biofilm formation.

ᵃ Microorganisms other than the experimentally inoculated A. baumannii were classified as non-target isolates and considered incidental microbiological findings.

Conventional cultures also yielded several non-target microorganisms, including *Klebsiella pneumoniae*, *Proteus mirabilis*, *Proteus vulgaris*, *Enterobacter cloacae*, *Escherichia coli*, *Klebsiella oxytoca*, *Staphylococcus aureus*, and *Lactobacillus* spp. These isolates were classified as non-target findings and were not included in the primary comparative analysis, which focused on recovery of the experimentally inoculated *A. baumannii* strain. Semiquantitative bacterial growth among culture-positive specimens ranged from 10^4^ to 10^7^ CFU.

Because microbiological assessment was limited to conventional culture, these findings indicate only whether viable cultivable microorganisms were recovered under the applied conditions. They do not provide quantitative information regarding bacterial burden, confirm biofilm formation or absence, or demonstrate complete bacterial clearance or antimicrobial activity of either implant material.

### 3.3 Tissue necrosis and implant degradation

**Figure 1:**
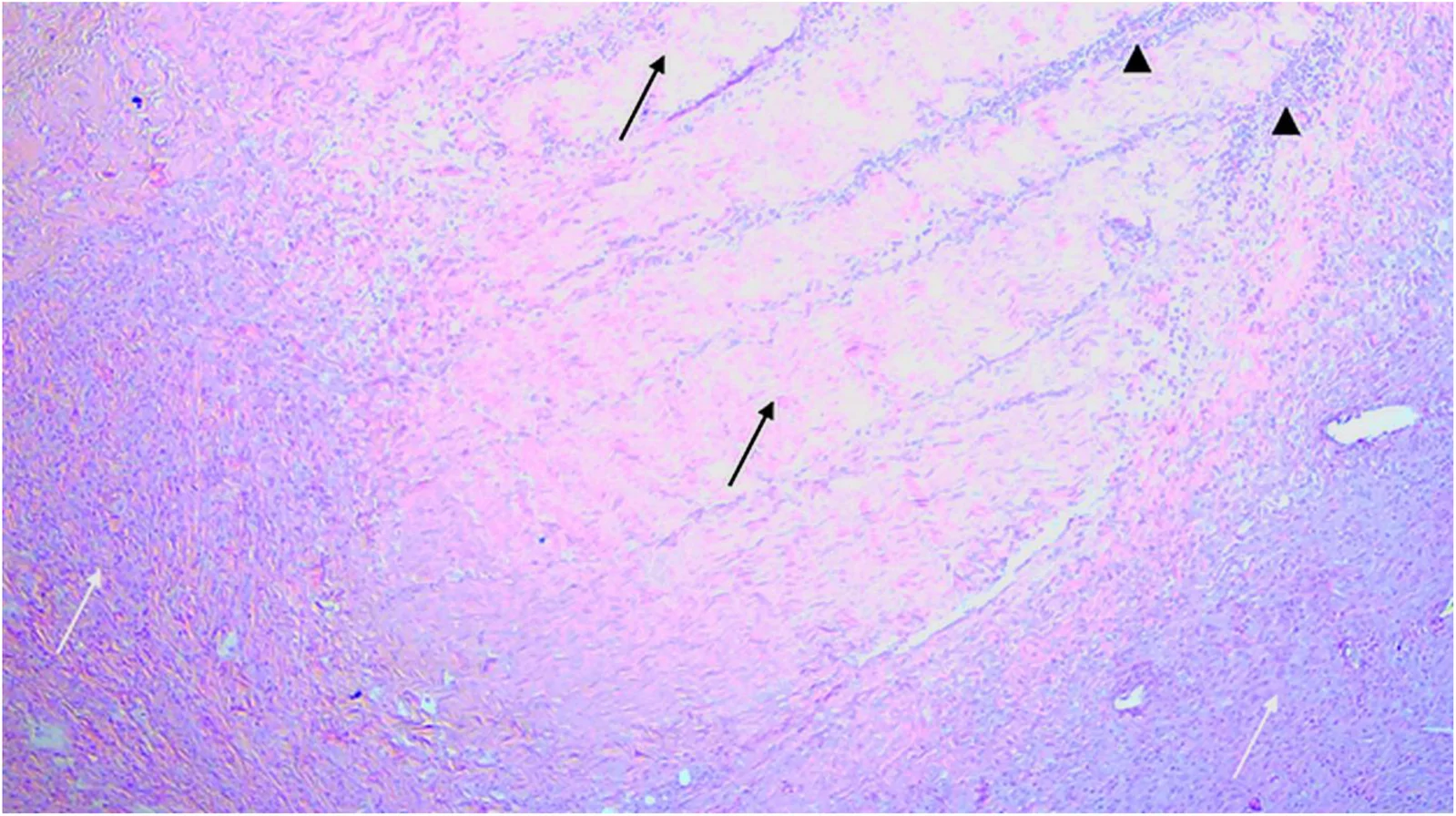
ECM implant under *A. baumannii* contamination on postoperative day 10 (Specimen No. 13/24). Hematoxylin and eosin stain, ×40. Fragments of the implanted xenoperitoneal matrix (black arrows) demonstrate focal inflammatory cell infiltration (arrowheads) and adjacent areas of early granulation tissue formation (white arrows).

On postoperative day 10, necrosis scores were significantly higher in the ECM group than in the UltraPro group (3.0 [0.75] vs. 1.0 [0.0]; *p* = 0.0049, *q* = 0.0234). Histological examination of ECM specimens demonstrated focal tissue disruption adjacent to fragments of the xenoperitoneal matrix, accompanied by inflammatory-cell infiltration and early granulation tissue formation (Figure 1).

**Figure 2:**
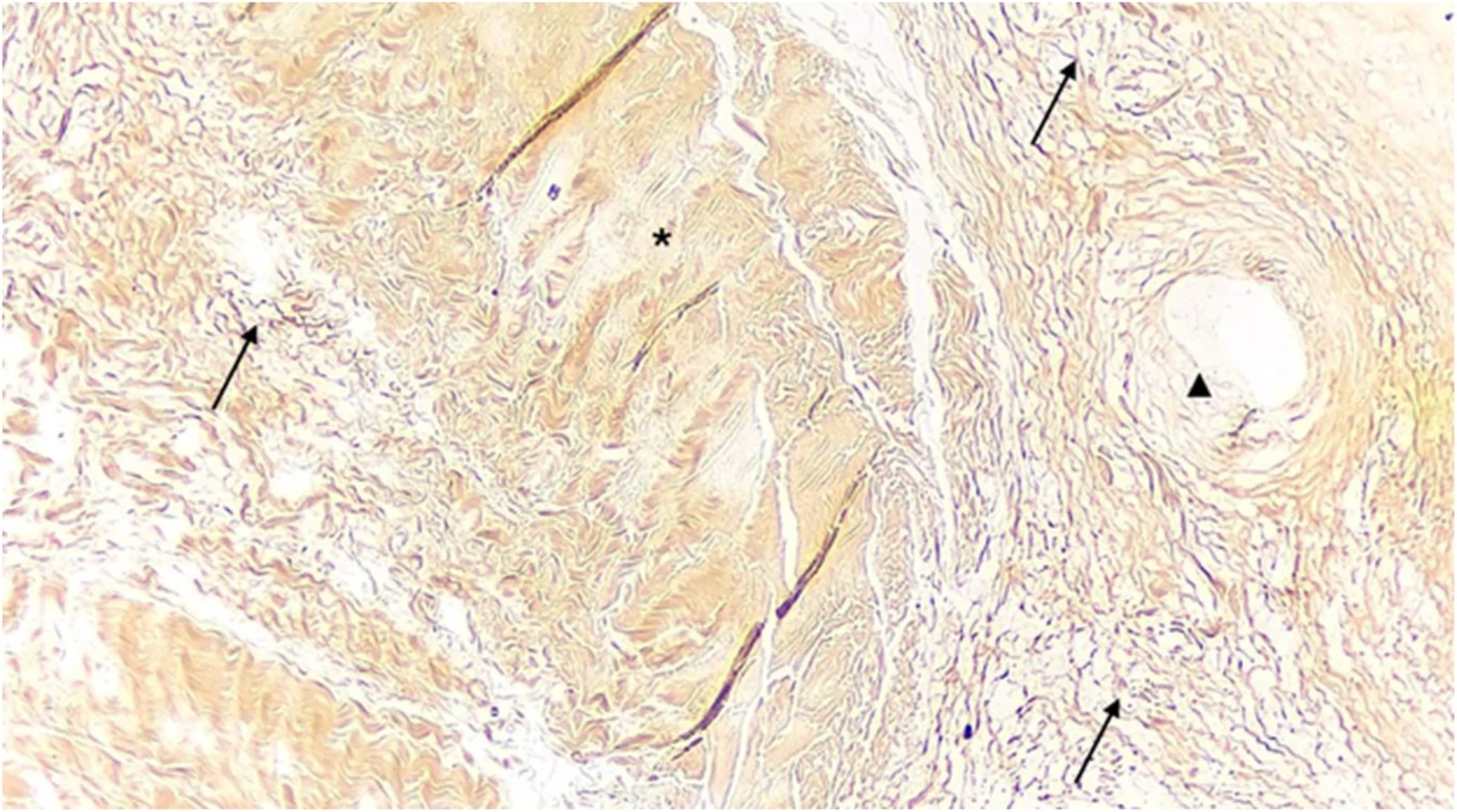
ECM implant under *A. baumannii* contamination on postoperative day 20 (Specimen No. 20/24). Gomori silver stain, ×100. At the implant–tissue interface, remodeled connective tissue (right side of the micrograph) demonstrates newly formed reticulin fibers (black arrows) surrounding suture material (arrowhead). Broad, wavy collagen bundles within the remodeled xenoperitoneal matrix are indicated by an asterisk.

By postoperative day 20, the median necrosis score in the ECM group had decreased from 3.0 [0.75] to 2.0 [0.0]. Histological sections demonstrated less extensive tissue disruption and more prominent cellular ingrowth and connective tissue formation at the implant–tissue interface than on postoperative day 10 (Figure 2). Nevertheless, necrosis remained significantly more pronounced in the ECM group than in the UltraPro group on day 20 (2.0 [0.0] vs. 1.0 [0.0]; *p* = 0.0067, *q* = 0.0234).

**Figure 3:**
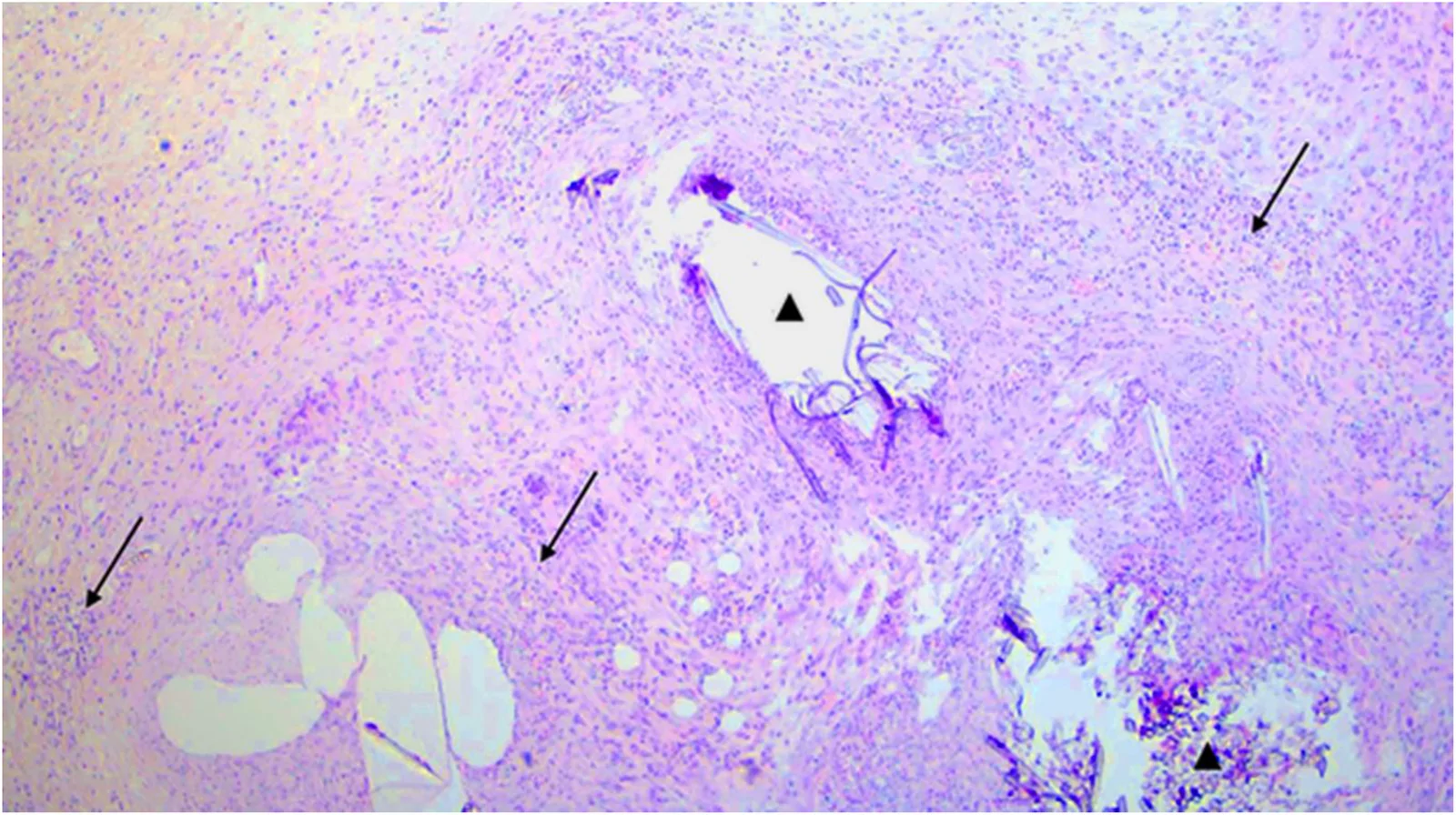
UltraPro mesh under *A. baumannii* contamination on postoperative day 10 (Specimen No. 52/24). Hematoxylin and eosin stain, ×40. Early connective tissue proliferation surrounding mesh filaments (black arrows) is accompanied by sparse inflammatory cell infiltration (arrowheads), consistent with an early foreign-body response.

In the UltraPro group, median necrosis scores were 1.0 [0.0] at both observation time points. No statistically significant temporal difference was detected (*p* = 0.405). On postoperative day 10, the surrounding tissue architecture was relatively preserved, with limited inflammatory changes around the mesh filaments (Figure 3).

Implant degradation scores in the ECM group decreased from 2.0 [0.75] on day 10 to 1.0 [0.0] on day 20; however, this difference did not reach statistical significance (*p* = 0.112). In the UltraPro group, the median score increased from 1.0 [0.0] to 1.5 [1.0], but the temporal difference was also not statistically significant (*p* = 0.282). Given the small subgroup size, these non-significant findings should not be interpreted as evidence of equivalence between the observation periods.

### 3.4 Polymorphonuclear cells

Polymorphonuclear (PMN) cell infiltration was significantly more pronounced in the ECM group than in the UltraPro group at both observation time points. On postoperative day 10, the median PMN score was 2.5 [1.0] in the ECM group and 1.0 [0.0] in the UltraPro group (*p* = 0.0024, *q* = 0.0233). In ECM specimens, inflammatory cells were concentrated around fragments of the implanted matrix and within areas of developing granulation tissue (Figure 1).

On postoperative day 20, PMN infiltration remained significantly higher in the ECM group than in the UltraPro group (2.0 [0.0] vs. 1.0 [0.0]; *p* = 0.0013, *q* = 0.0233). Although the median PMN score in the ECM group was lower than on day 10, inflammatory cells remained present adjacent to the implanted matrix, particularly at the implant–tissue interface.

In the UltraPro group, median PMN scores were 1.0 [0.0] at both observation time points, and no statistically significant temporal difference was detected. Histological examination demonstrated sparse inflammatory cells, predominantly localized around the mesh filaments during the early postoperative period (Figure 3).

### 3.5 Lymphocytes

Lymphocytic infiltration increased significantly in the ECM group between postoperative days 10 and 20, from 1.0 [0.0] to 2.0 [0.75] (*p* = 0.0063, *q* = 0.0234). On day 10, lymphocytes were observed focally within areas of inflammatory infiltration. By day 20, lymphocytic infiltration was more pronounced, particularly at the implant–tissue interface and in areas of cellular colonization of the matrix (Figure 2).

In the UltraPro group, median lymphocyte scores were 2.0 [0.0] at both observation time points, with no statistically significant temporal difference. Histological examination demonstrated lymphocytic infiltration surrounding the mesh filaments, without marked differences in its distribution between days 10 and 20.

Between-group differences in lymphocyte scores were not statistically significant at either observation time point.

### 3.6 Multinucleated giant cells

Multinucleated giant-cell scores in the ECM group were 1.0 [0.0] at both postoperative days 10 and 20, with no statistically significant temporal difference. Histological examination revealed only occasional giant cells without clustering or a pronounced foreign-body reaction.

**Figure 4:**
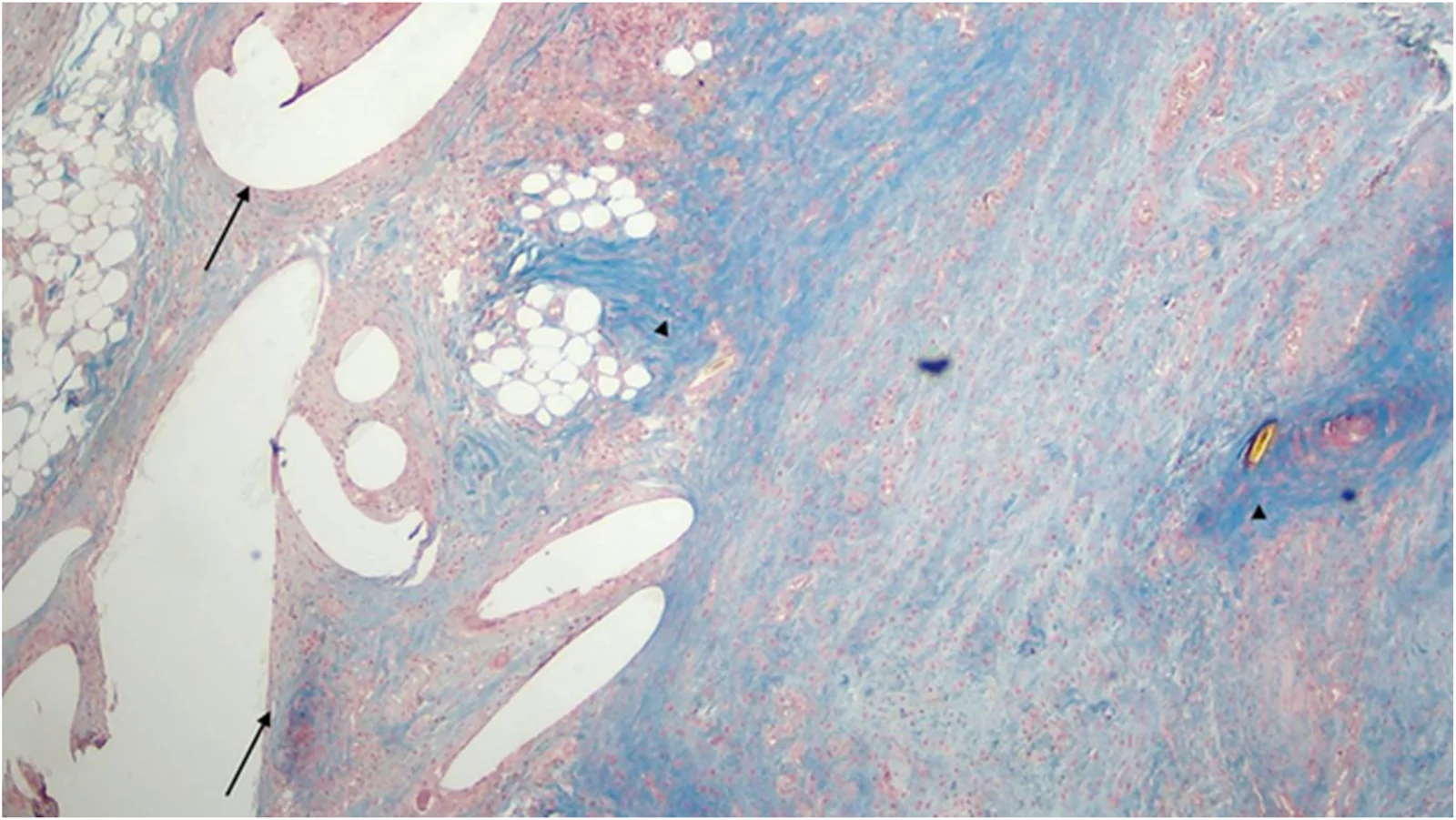
UltraPro mesh under *A. baumannii* contamination on postoperative day 20 (Specimen No. 59/24). Masson's trichrome stain, ×40. Marked connective tissue proliferation surrounding mesh filaments (black arrows) is associated with progressive fibrotic encapsulation, represented by dense dark-blue collagen bundles (arrowheads). Areas of loose collagen deposition intermixed with cellular infiltration are also observed.

In the UltraPro group, multinucleated giant-cell scores increased significantly from 1.0 [0.0] on day 10 to 2.5 [1.0] on day 20 (*p* = 0.0067, *q* = 0.0234). On postoperative day 10, isolated giant cells were observed adjacent to the mesh filaments, accompanied by sparse inflammatory-cell infiltration (Figure 3). By day 20, multinucleated giant cells were more prominent and frequently surrounded the mesh filaments, together with granulomatous changes and increased connective tissue proliferation (Figure 4).

On postoperative day 20, multinucleated giant-cell scores were significantly higher in the UltraPro group than in the ECM group (2.5 [1.0] vs. 1.0 [0.0]; *p* = 0.0025, *q* = 0.0233).

### 3.7 Neovascularization, fibrosis, and granulomas

Median neovascularization scores in the ECM group were 1.0 [0.0] at both observation time points. UltraPro specimens demonstrated significantly higher neovascularization scores than ECM specimens on postoperative day 10 (2.0 [0.75] vs. 1.0 [0.0]; *p* = 0.0081, *q* = 0.0234) and day 20 (2.0 [0.0] vs. 1.0 [0.0]; *p* = 0.0018, *q* = 0.0233). Newly formed vessels were observed within areas of connective tissue proliferation surrounding the mesh filaments.

Median fibrosis scores in the ECM group were 1.0 [0.0] at both observation time points. In the UltraPro group, fibrosis scores increased from 2.0 [1.0] on day 10 to 3.0 [0.75] on day 20. The temporal difference was significant before correction for multiple comparisons (*p* = 0.0225) but did not remain among the statistically significant comparisons after FDR correction. Fibrosis scores were significantly higher in the UltraPro group than in the ECM group on postoperative day 10 (*p* = 0.0089, *q* = 0.0234) and day 20 (*p* = 0.0089, *q* = 0.0234). On day 20, histological examination demonstrated dense collagen deposition surrounding the mesh filaments and fibrous capsule formation (Figure 4).

Granulomas were not identified in ECM specimens at either observation time point (0/6). In the UltraPro group, granulomatous inflammation was identified in 2 of 6 specimens (33.3%) on day 10 and 5 of 6 specimens (83.3%) on day 20. These descriptive findings were accompanied by connective tissue proliferation and inflammatory-cell accumulation around the mesh filaments (Figure 4).

### 3.8 Matrix colonization and extracellular fiber deposition

Cellular colonization of the implanted ECM increased from 1.5 [1.0] on postoperative day 10 to 3.0 [1.0] on day 20 (*p* = 0.0080, *q* = 0.0234). On day 10, cellular ingrowth within the xenoperitoneal matrix was focal and limited. By day 20, cells occupied larger areas of the implanted material, representing histological features consistent with early tissue organization at the implant–tissue interface.

Reticulin fiber scores in the ECM group also increased between days 10 and 20, from 1.0 [0.0] to 2.0 [0.75] (*p* = 0.0081, *q* = 0.0234). Gomori silver staining demonstrated newly formed reticulin fibers adjacent to the implanted matrix, particularly within areas of cellular ingrowth and connective tissue organization (Figure 2). On day 20, reticulin fiber scores were significantly higher in the ECM group than in the UltraPro group (2.0 [0.75] vs. 1.0 [0.0]; *p* = 0.0081, *q* = 0.0234).

Cellular colonization of the implant matrix was not applicable to UltraPro mesh. Reticulin fiber scores in the UltraPro group were 1.0 [0.0] at both observation time points, with no statistically significant temporal difference (*p* = 1.000). Elastic fibers were evaluated only in UltraPro specimens; their median score increased from 1.0 [0.75] to 1.5 [1.0], but the difference was not statistically significant (*p* = 0.523).

In the UltraPro group, fat infiltration scores increased from 2.0 [0.75] on day 10 to 3.0 [0.75] on day 20. This difference was significant before correction for multiple comparisons (*p* = 0.0225) but did not remain significant after FDR correction. Capsule formation scores increased from 1.0 [0.0] to 2.5 [1.0], although the temporal difference was not statistically significant (*p* = 0.306). On day 20, histological examination demonstrated connective tissue accumulation and fibrous encapsulation around the mesh filaments (Figure 4).

The principal comparisons that remained statistically significant after FDR correction are summarized in Table 5.

**Table 5:** Main statistically robust histomorphometric comparisons after FDR correction.

Comparison	Parameter	*p*-value	FDR-adjusted q-value
ECM vs UltraPro, day 10	Necrosis	0.0049	0.0234
ECM vs UltraPro, day 10	Polymorphonuclear cells	0.0024	0.0233
ECM vs UltraPro, day 10	Neovascularization	0.0081	0.0234
ECM vs UltraPro, day 10	Fibrosis	0.0089	0.0234
ECM vs UltraPro, day 20	Necrosis	0.0067	0.0234
ECM vs UltraPro, day 20	Polymorphonuclear cells	0.0013	0.0233
ECM vs UltraPro, day 20	Multinucleated giant cells	0.0025	0.0233
ECM vs UltraPro, day 20	Neovascularization	0.0018	0.0233
ECM vs UltraPro, day 20	Fibrosis	0.0089	0.0234
ECM vs UltraPro, day 20	Reticulin fibers	0.0081	0.0234
ECM, day 10 vs day 20	Lymphocytes	0.0063	0.0234
ECM, day 10 vs day 20	Cellular colonization of matrix	0.0080	0.0234
ECM, day 10 vs day 20	Reticulin fibers	0.0081	0.0234
UltraPro, day 10 vs day 20	Multinucleated giant cells	0.0067	0.0234

## 4 Discussion

The 20-day observation period allowed assessment of early and subacute tissue responses but was insufficient to evaluate mature fibrosis, complete ECM remodeling, long-term scaffold degradation, biomechanical durability, or functional abdominal wall reconstruction. Under experimental *A. baumannii* contamination, ECM and UltraPro demonstrated distinct early histological response patterns. These changes occurred in the absence of clinically evident wound infection, dehiscence, abscess formation, or mortality.

On postoperative day 10, ECM specimens showed greater necrosis and polymorphonuclear-cell infiltration than UltraPro specimens. This early response may reflect the combined effects of bacterial contamination and the initial host reaction to a degradable biologic scaffold. Decellularized matrices can recruit inflammatory and stromal cells during the early phase after implantation, contributing to degradation of matrix components and subsequent cellular repopulation (2, 25). However, the relatively high necrosis scores should not be interpreted as evidence of either constructive remodeling or implant intolerance. Because bacterial burden and biofilm formation were not quantified, the relative contributions of the implanted material and *A. baumannii* contamination cannot be determined.

By postoperative day 20, ECM specimens demonstrated increased lymphocytic infiltration, cellular colonization of the implanted matrix, and reticulin fiber deposition. These changes are consistent with early tissue organization at the implant–tissue interface. Nevertheless, they do not establish complete ECM remodeling, mature regeneration, stable integration, or adequate biomechanical strength. Longer observation periods are required to determine whether these early morphological changes progress toward mature collagen organization and durable functional incorporation.

Immune modulation may contribute to the response of decellularized ECM scaffolds. Previous studies have linked constructive dECM remodeling to interactions among macrophages, regulatory T cells, and other components of the local immune microenvironment (26, 27). Changes in macrophage phenotype may influence inflammation, scaffold degradation, cellular ingrowth, and connective tissue deposition. However, macrophage phenotyping, immunohistochemical assessment of M1- or M2-associated markers, cytokine profiling, and regulatory T-cell analysis were not performed in the present study. Therefore, the observed cellular colonization and reticulin deposition cannot be attributed to a specific macrophage phenotype or immunomodulatory pathway.

The non-contaminated ECM observations presented in Table 3 provide descriptive context only. In that independent experimental series, granulocytic infiltration decreased over time, whereas fibroblast density and connective tissue maturation increased. However, these observations were obtained on postoperative days 7, 14, and 21 in a separate experiment, and no corresponding non-contaminated UltraPro group was available. They cannot replace concurrent sterile implant controls or determine whether the findings in the present study resulted from the implant material, bacterial contamination, or an interaction between these factors.

The current findings are broadly consistent with previous experimental studies of xenoperitoneal ECM, which described cellular ingrowth and connective tissue organization after implantation (22, 23, 28–30). In the present study, cellular colonization and reticulin deposition increased by day 20. These observations support the presence of early structural changes within the implanted matrix but should not be interpreted as proof of favorable long-term remodeling or superior integration. Furthermore, the observed differences in fibrosis, reticulin deposition, and neovascularization represent semi-quantitative histological scores and should not be interpreted as objective measurements of collagen amount, collagen organization, fiber orientation, or vascular density.

UltraPro demonstrated a different response pattern. Neovascularization and fibrosis scores were higher than those of ECM at both observation time points. Multinucleated giant-cell activity also increased significantly between days 10 and 20. Granulomatous inflammation and capsule formation were more frequently observed by day 20, although some of these temporal changes did not remain statistically significant after FDR correction and should therefore be interpreted descriptively.

Multinucleated giant cells are a characteristic component of the foreign-body response to implanted biomaterials. Their formation involves macrophage fusion and is influenced by inflammatory signals, material properties, and macrophage–fibroblast interactions (25, 31). In the present study, giant cells were increasingly observed around UltraPro filaments together with connective tissue proliferation. However, their presence does not independently establish implant failure, and the short observation period does not permit conclusions regarding mature encapsulation, mesh contraction, or long-term functional consequences.

Bacterial contamination may also have contributed to the observed inflammatory and foreign-body responses. *A. baumannii* can survive on abiotic surfaces, adhere to biomaterials, and form biofilms, potentially prolonging inflammation and impairing tissue integration (9, 10, 16, 17, 32). Nevertheless, the current design cannot distinguish material-specific responses from the effects of bacterial persistence because concurrent sterile ECM and UltraPro groups were not included.

Conventional culture did not recover viable *A. baumannii* from ECM-associated specimens, whereas the organism was recovered from 2 of 12 UltraPro-associated specimens. These findings should be interpreted strictly as culture-based observations. Quantitative bacterial burden, bacterial DNA, and biofilm formation were not assessed. Therefore, the absence of culture-detectable *A. baumannii* in ECM specimens does not demonstrate complete bacterial clearance, reduced bacterial persistence, biofilm inhibition, or intrinsic antimicrobial activity of the ECM scaffold. Similarly, the study cannot determine whether the histological differences were related to bacterial burden, material-specific host responses, or both.

The results should also be considered in the context of clinical evidence. Randomized trials of contaminated ventral hernia repair have not demonstrated clinical superiority of biologic mesh and have reported lower recurrence rates with synthetic mesh in some settings (7, 8). The present short-term rat study does not contradict these clinical findings because it evaluated early local histological responses rather than recurrence, mechanical strength, cost, or long-term clinical outcomes.

These findings should be interpreted in the context of the exploratory study design and the small subgroup size (*n* = 6). Although several comparisons remained statistically significant after false discovery rate correction, the limited number of animals reduces the precision and robustness of the observed estimates. Conversely, non-significant comparisons cannot exclude potentially biologically relevant differences and should not be interpreted as evidence of equivalence.

Accordingly, the present findings are primarily mechanistic and hypothesis-generating. ECM and UltraPro demonstrated different early tissue-response patterns under standardized *A. baumannii* contamination, but the study does not establish superior antimicrobial, biomechanical, functional, or clinical performance of either material. Future studies should use concurrent sterile and contaminated ECM and UltraPro groups, longer follow-up, quantitative bacterial burden expressed as CFU/g of tissue, direct biofilm visualization using scanning electron or confocal microscopy, molecular bacterial detection methods including bacterial DNA quantification by PCR, and standardized digital image analysis of collagen area fraction, collagen density, fiber orientation, and vascular density, together with macrophage phenotyping and biomechanical testing.

### 4.1 Strengths and limitations

This study has several strengths, including a standardized contamination model, direct comparison of biologic and synthetic implants under identical experimental conditions, blinded histopathological assessment, multiple histological staining methods, and semi-quantitative evaluation based on ISO 10993-6 principles.

Several limitations should be acknowledged. The 20-day observation period allowed assessment only of early and subacute tissue responses and did not permit evaluation of complete ECM remodeling, mature fibrosis, long-term implant integration, biomechanical durability, or functional abdominal wall reconstruction. Mechanical testing was outside the predefined scope of this morphological study.

Microbiological assessment was limited to conventional culture. Quantitative bacterial burden, bacterial DNA, and direct biofilm visualization were not assessed; therefore, the findings cannot establish bacterial clearance, quantify persistence, or determine whether the histological differences were related to bacterial burden or material-specific host responses. Histomorphometric evaluation of fibrosis, reticulin deposition, and neovascularization was semi-quantitative. Objective digital measurements of collagen area fraction, collagen density, fiber orientation, and vascular density were not performed. Because standardized whole-slide acquisition, uniform field selection, image calibration, and a prospectively defined digital morphometry protocol were not used, retrospective quantitative analysis of the available illustrative micrographs could introduce selection and measurement bias.

Each subgroup included six animals, and no formal *a priori* power calculation was performed. Although FDR correction was applied, the small sample size limited statistical power and the precision of the findings. Non-significant results should not be interpreted as evidence of equivalence, and all statistical findings should be considered exploratory. Clinical observations were recorded descriptively without formal wound-infection or pain/distress scoring systems.

Finally, the absence of concurrent sterile ECM and sterile UltraPro implantation groups represents a major limitation of the experimental design. Consequently, the study cannot distinguish material-specific tissue responses from the effects of bacterial contamination or determine whether an interaction occurred between implant type and contamination. The non-contaminated ECM observations presented in Table 3 originated from an independent experimental series and provide descriptive context only. They cannot replace simultaneous sterile control groups, particularly because the observation periods differed and no non-contaminated UltraPro group was available. A future factorial experimental design including sterile ECM, contaminated ECM, sterile UltraPro, and contaminated UltraPro groups evaluated at identical postoperative time points is required to address these questions.

### 4.2 Clinical implications

The present findings provide preclinical information on early tissue responses to biologic and synthetic implants under experimental *A. baumannii* contamination. ECM demonstrated histological features consistent with early tissue organization, whereas UltraPro showed more pronounced foreign-body and fibrotic responses during the observation period. However, these morphological findings do not establish superior infection resistance, biomechanical performance, or clinical effectiveness of either material.

Therefore, the results should be used primarily to inform the design of further preclinical studies rather than clinical implant selection. Translation to clinical practice requires longer follow-up, quantitative microbiological and biofilm assessment, biomechanical testing, functional evaluation, and further development of implant materials capable of limiting bacterial adhesion and biofilm formation (33). Clinical decisions should continue to consider the degree of surgical-field contamination, recurrence risk, and the potential for postoperative infectious complications when selecting an implant material (34).

## 5 Conclusions

This study demonstrated distinct early histological response patterns to bovine-derived peritoneum ECM and UltraPro mesh under experimental *A. baumannii* contamination. ECM was associated with a more pronounced acute inflammatory response, followed by reduced necrosis, increased cellular colonization, and greater reticulin fiber deposition by postoperative day 20—findings consistent with early tissue organization. UltraPro demonstrated increasing multinucleated giant-cell activity, fibrosis, granulomatous inflammation, and capsule formation during the observation period.

Given the small subgroup size, short follow-up, absence of concurrent sterile implant controls, and lack of quantitative microbiological and biomechanical assessment, these findings should be considered exploratory and do not establish bacterial clearance or superior functional or clinical performance of either material. The separate non-contaminated ECM observations provide contextual descriptive information only and do not compensate for the absence of concurrent sterile ECM and UltraPro control groups. Further studies incorporating longer follow-up, quantitative microbiology, biofilm assessment, mechanical testing, and functional outcomes are required.

## Data Availability

The original contributions presented in the study are included in the article/Supplementary Material, further inquiries can be directed to the corresponding author/s.
